# Effectiveness and safety of a home-based cardiac rehabilitation programme of mixed surveillance in patients with ischemic heart disease at moderate cardiovascular risk: A randomised, controlled clinical trial

**DOI:** 10.1186/s12872-017-0499-0

**Published:** 2017-02-20

**Authors:** Raquel Bravo-Escobar, Alicia González-Represas, Adela María Gómez-González, Angel Montiel-Trujillo, Rafael Aguilar-Jimenez, Rosa Carrasco-Ruíz, Pablo Salinas-Sánchez

**Affiliations:** 1 Unidad de Rehabilitación Cardiaca, Hospital universitario Virgen de la Victoria de Málaga, Campus de Teatinos s/n, 29010 Málaga, España; 2 Facultad de Fisioterapia, Departamento de Biología Funcional y Ciencias de la Salud, Universidad de Vigo, Campus A Xunqueira s/n, 36005 Pontevedra, España; 3 Servicio de Cardiología, Hospital universitario Virgen de la Victoria de Málaga, Campus de Teatinos s/n, 29010 Málaga, España; 4 Facultad de Medicina, Departamento de Anatomía Humana, Medicina legal e Historia de la Ciencia, University of Málaga, Campus de Teatinos s/n, 29010 Malaga, España

**Keywords:** Telemedicine, Rehabilitation, Exercise therapy, Myocardial ischemia

## Abstract

**Background:**

Previous studies have documented the feasibility of home-based cardiac rehabilitation programmes in low-risk patients with ischemic heart disease, but a similar solution needs to be found for patients at moderate cardiovascular risk. The objective of this study was to analyse the effectiveness and safety of a home-based cardiac rehabilitation programme of mixed surveillance in patients with ischemic cardiopathology at moderate cardiovascular risk.

**Methods:**

A randomised, controlled clinical trial was designed wherein 28 patients with stable coronary artery disease at moderate cardiovascular risk, who met the selection criteria for this study, participated. Of these, 14 were assigned to the group undergoing traditional cardiac rehabilitation in hospital (control group) and 14 were assigned to the home-based mixed surveillance programme (experimental group). The patients in the experimental group went to the cardiac rehabilitation unit once a week and exercised at home, which was monitored with a remote electrocardiographic monitoring device (NUUBO®). The in-home exercises comprised of walking at 70% of heart rate reserve during the first month, and 80% during the second month, for 1 h per day at a frequency of 5 to 7 days per week. A two-way repeated measures analysis of variance (ANOVA) was performed to evaluate the effects of time (before and after intervention) and time-group interaction regarding exercise capacity, risk profile, cardiovascular complications, and quality of life.

**Results:**

No significant differences were observed between the traditional cardiac rehabilitation group and the home-based with mixed surveillance group for exercise time and METS achieved during the exertion test, and the recovery rate in the first minute (which increased in both groups after the intervention). The only difference between the two groups was for quality of life scores (10.93 [IC95%: 17.251, 3.334, *p* = 0.007] vs −4.314 [IC95%: −11.414, 2.787; *p* = 0.206]). No serious heart-related complications were recorded during the cardiac rehabilitation programme.

**Conclusions:**

The home-based cardiac rehabilitation programme with mixed surveillance appears to be as effective and safe as the traditional model in patients with ischemic heart disease who are at moderate cardiovascular risk. However, the cardiac rehabilitation programmes carried out in hospital seems to have better results in improving the quality of life.

**Trial registration:**

Retrospectively registered NCT02796404 (May 23, 2016).

## Background

Cardiovascular diseases are the main causes of mortality in today’s society and the primary cause of hospital admissions. They are responsible for up to 30% of all deaths worldwide and 48% of deaths in Europe, and it is expected that these figures will increase in the coming years [1].

Cardiac rehabilitation is not a new concept. Various studies have shown the effectiveness of these programmes in reducing cardiac mortality [2–4] by between 27 and 31% in patients with coronary heart disease; unfortunately, participation levels in these programmes continue to be low in some countries [1]. Sociodemographic factors, factors related to programme availability and accessibility, and the low referral rates of patients to this service are associated with lower rates of patient participation in cardiac rehabilitation programmes [5, 6].

All of these challenges have led to the recent development of alternative models of cardiac rehabilitation, with innovative approaches to improve the delivery and use of these programmes and to provide solutions to these barriers [7].

The effectiveness of home-base cardiac rehabilitation/secondary prevention programs is not new [8]. Studies carried out in the 1980’s documented the feasibility of home-based cardiac rehabilitation programmes in low-risk patients with ischaemic cardiomyopathy [9]. While some authors maintained that such programmes provided a solution to the low patient participation in rehabilitation programmes [9], a solution also needs to be found for moderate-risk patients so that they can follow a safe and effective home-based cardiac rehabilitation programme.

Implementing telemedicine in cardiac rehabilitation through the use of technology could partially reduce the logistical problems described above. Telemonitoring devices can allow the patient to be monitored at home so that the patient does not have to travel, and they can improve the efficiency of health care processes by incorporating this information in a comprehensive management programme for cardiac patients [10, 11].

As with any other form of health technology, the effectiveness and safety of these devices must be assessed before their use is extended [10]. In this sense, some alternative home-based cardiac rehabilitation/secondary prevention program with remote monitoring has been previously described [12], but clinical trials evaluating the efficacy of these programs in patients with high risk, greater comorbidity, and also with stable angina are necessary [8].

If home-based cardiac rehabilitation programmes with telemonitoring offer the same safety and effectiveness in moderate-risk patients, it could herald a change in health insurance policies to extend the coverage of these programmes. This would allow a greater number of patients to access cardiac rehabilitation programmes without increasing the total costs [6].

Very few studies have evaluated a home-based cardiac rehabilitation programme model with mixed surveillance, and the studies to date have focused on patients with a low-risk profile. For this reason, it was necessary to develop an alternative home-based cardiac rehabilitation model aimed at higher-risk patients.

The objective of this study was to analyse the effectiveness and safety of a home-based cardiac rehabilitation programme with mixed surveillance in moderate-risk patients with ischaemic cardiomyopathy.

## Methods

### Study population

Patients with stable ischaemic cardiomyopathy who had undergone revascularisation by either stent-angioplasty or by-pass surgery, who were at moderate risk according to the Spanish Cardiology Society’s clinical practice guidelines (Table 1) [8, 13–17], aged ≤ 75, with a good cognitive level, a capacity to perform aerobic exercise on a treadmill or stationary bike, and knowledge of how to use a smartphone or tablet were included in this study. Patients also had to meet at least one of the following inclusion criteria: ventricular dysfunction using ejection fraction (EF) 40–55%, functional capacity 5–7 METS, and/or raised blood pressure with exertion.

**Table 1:** Classification criteria

Risk	Clinical Variables	Complementary Tests	Physical Capacity
Low	Age <50 years Clinical course with no complications. Killip I No previous infarct. Asymptomatic.	No signs of ischemia. Ejection fraction >50%. Normal BP response to exercise. No arrhythmias.	≥7 METS
Moderate	Age >50 years Killip I or II No previous infarct. Mild symptomology.	Effort angina or mild signs of ischemia. Ejection fraction between 35-49%. Mild BP elevation with effort. Low grade arrhythmias. Reversible thallium defect with effort.	>5 METS
High	Killip II or III Previous infarct. Symptoms with low load.	Severe ischemia. ST depression of >2 mm at a rate of less than 135 beats per minute. Hypertensive response to effort. Malignant arrhythmias.	<5 METS

We excluded patients with malignant arrhythmias (ventricular fibrillation >24 h after the acute myocardial infarction, ventricular tachycardia, grade 2 or 3 AV block, atrial fibrillation in patients with Wolff-Parkinson-White syndrome, fibrillation or paroxysmal flutter with rapid ventricular response and haemodynamic deterioration, ventricular extrasystole which increases during exertion and uncontrolled supraventricular tachycardia), previous infarctions, ischemia induced by exercise, unstable angina, disease not amenable to revascularisation, poorly-controlled high blood pressure, associated valvular heart disease, pacemaker or ICD-CRT and locomotor system, or a neurological or respiratory pathology that would make walking for prolonged periods difficult.

The study protocol complied with the Declaration of Helsinki and was reviewed and approved by the Comité de Ética Interprovincial de Málaga [Malaga Interprovincial Ethics Committee] belonging to the Consejería de Salud [Health Council] of the Junta de Andalucía [Regional Government of Andalusia] and registered in the clinical trials register (NCT02796404). The study met the requirements of the Andalusian Health System order SAS 3470/2009 and with Organic Law 15/1999 of 13 December for the Protection of Data of a personal nature.

The participants received information about the study methods and gave their written informed consent.

### Study design

This is an ongoing randomised controlled multicentre clinical trial (NCT02796404). In this paper, we present preliminary data from one of the participating centres, the Hospital Virgen de la Victoria in Malaga (Spain).

The patients were assigned randomly by an independent investigator to either the control group, which participated in the usual cardiac rehabilitation programme at the hospital, or to the experimental group, which participated in the home-based mixed surveillance programme. The software program Epidat was used for the randomization, ensuring a balanced distribution in both groups. The algorithm generated by this program established that every time a new subject was assigned to a particular group, the probability that that subject was assigned to that group would be inversely proportional to the number of individuals included in it up to that point.

**Fig. 1:**
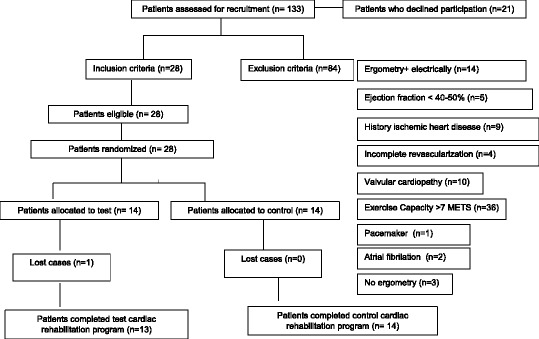
Flowchart of the study design

The cardiac rehabilitation programme in both cases had a duration of 2 months. There was only one patient who withdrew from the home-based mixed surveillance group due to personal reasons. The outline design is shown in Fig. 1.

### Intervention

The two cardiac rehabilitation programs were started 4–6 months after the patients were discharged from the hospital. Both programs included physical exercise, health education, and physiotherapy, with a duration of 2 months in both cases.

The usual cardiac rehabilitation programme took place in the cardiac rehabilitation unit of the Hospital Virgen de la Victoria in Malaga (Spain). The patients in this group attended the hospital exercise programme 3 times a week (24 sessions) and were encouraged to do exercise at home according to the European Society of Cardiology guidelines [18].

The patients in the home-based cardiac rehabilitation with mixed surveillance group only went to the cardiac rehabilitation unit once a week. During this visit, they had a supervised physical exercise session that was identical to the usual cardiac rehabilitation session. Then they exercised at home following a walking program for 1 h in duration at 70% of the reserve heart rate following the Karvonen formula during the first month and 80% during the second, which was monitored with a remote electrocardiographic monitoring device NUUBO®, for at least two more days a week, although they were encouraged to exercise every day.

The NUUBO® system uses Bluetooth wireless technology and biometric vests using textile electrodes developed and patented by Nuubo, BlendFix Sensor Electrode Technology, which makes its use simple. On the first day, the patients were instructed on how to use this device’s mobile application and they were all given a smartphone with Internet connection and the NUUBO® application pre-installed. Each patient was registered on the server; clinical details were entered along with the previously calculated and adjusted exercise heart rate and exercise time (60 min).

The exercise program prescribed for both groups consisted of 15 min of warm-up exercise (stretching and isotonic exercises), followed by 30 min of continuous aerobic exercise, alternating between treadmill training 1 day and stationary bike training the next day, at 70% of the reserve heart rate, following the Karvonen formula during the first month and 80% during the second. The intensity of the exercise was calculated using the heart rate value obtained during the stress test. The patients ended the exercise session with a 15-min cool-down period. Once a week the patients also did a prescribed strength-training session consisting of one or two series of 10 repetitions of exercise for the brachial biceps, brachial triceps, pectoris major, deltoids, and quadriceps at 20 MR (maximum resistance) that is equivalent to 50% of 1MR, with a recovery time of 2–3 min recovery time between each set of repetitions.

Both groups had a health education session at the hospital once a week to improve awareness and understanding about subjects related to the anatomy and function of the heart, cardiovascular risk factors, physical exercise, medication, diet, erectile dysfunction, and returning to work.

All of the patients also attended a group psychotherapy support and assessment session once a week to reduce the emotional impact of the disease, improve their state of health, reduce their chances of another infarction, help them accept their diagnosis, and improve their quality of life.

### Measurements

The following measurements were taken at the start of the study and at the end of the 2-months cardiac rehabilitation programmes.

#### Anthropometric measurements

The body weight of the patients was recorded using a calibrated scale. The measurements were taken with patients wearing light clothes and no shoes. The body mass index (BMI) was calculated based on the formula weight (kg)/height^2^ (m). The abdominal circumference was measured with a metric tape with the patient standing up with legs together, arms by the side, and abdomen relaxed after a deep breath. The circumference of the area located at mid-point between the costal edge and the upper edge of the iliac crest was then measured.

#### Blood pressure

Systolic and diastolic blood pressure readings were taken with an Omro M5-I ® electric sphygmomanometer. The measurements were taken after 10 min of rest with the arm on a table, and the average of three readings was recorded.

#### Exercise capacity

The participants took an exertion test before enrolling in the study to ensure that they could tolerate the exercise and to determine their METS and exercise time (minutes). The exertion test was done on a treadmill (General Electrics®, Marquette - CASE 8000 Exercise Testing System), following the clinical practice guidelines with continual monitoring with 12-derivation electrocardiogram, using the Bruce protocol. The test ended when the participants were exhausted or showed signs and/or symptoms of intolerance.

Maximum heart rate reached in the stress test was recorded. The heart rate recovery rate during the first minute and the perceived exertion level according to the Borg scale, RPE 6–20, were also recorded.

#### Laboratory parameters

The participant’s total cholesterol, HDL cholesterol, LDL cholesterol, triglycerides, blood glucose and glycated haemoglobin (HbA1c) were analysed in the Clinical Analysis Laboratory of the Laboratory and Haematology Clinical Management Unit of the Hospital Virgen de la Victoria. The glucose, cholesterol and triglycerides levels were recorded using the “Dimension Vista” measuring equipment from Siemens of Grunelwald, Germany. The HbA1c levels were determined using the Adams A1c, HA-8180 V analyser. The glucose levels were measured using the glucose hexokinase method. The total cholesterol levels were analysed using the cholesterol oxidase method, the HDL cholesterol levels were analysed with the enzymatic magnesium dextransulphate method, and the LDL cholesterol levels were analysed using the selective detergent method. Finally, the triglycerides levels were determined using the lipase/glycerol kinase method.

#### Assessment of health-related quality of life

The Medical Outcome Survey Short Form (SF-36) questionnaire was used to measure the quality of life. The SF-36 consists of 36 items which assess 8 different dimensions, and the possible scores range from 0–100; the variables assessed include limitations due to physical problems, body pain, social function or role, mental health, limitation due to emotional problems, vitality, energy or fatigue, and general perception of health. Studies published on the metric characteristics of the Spanish version of the SF-36 have provided enough evidence in support of its reliability, validity, and sensitivity [19].

#### Cardiovascular complications

Complications that arose during the hospital and home-based cardiac rehabilitation programmes were recorded, and they included AMI, severe arrhythmias, cardiorespiratory arrest or death, arterial hypotension, arterial hypertension, hypoglycaemia, hyperglycaemia, or intermittent claudication due to peripheral arterial disease.

### Statistical analysis

A two-way repeated measures analysis of variance (ANOVA) was carried out to evaluate the effect of the time (before and after intervention) and the effect of time-group interaction. The normality of distributions was verified using the Shapiro-Wilk test. Descriptive analyses were performed to characterise the sample and the study outcomes using the central tendency, confidence interval, and percentage measures. The level of significance was set at *p* < 0.05 for all tests.

## Results

A total of 28 patients initially participated in the study, all men with an average age of 56.07 (±8.92); 14 of the participants were assigned to the usual cardiac rehabilitation group (control group) and 14 were assigned to the home-based mixed surveillance programme (experimental group). During the intervention phase, one patient from the experimental group withdrew from the study for personal reasons. The initial characteristics of the sample are shown in Table 2. No significant differences were seen between both groups except for the number of patients presenting with diabetes mellitus and the maximum heart rate achieved in the stress test. The differences in this latter parameter probably can be related to the response to the medication of some patients since there are no differences in METS achieved nor in the time of exercise.

**Table 2:** Baseline characteristics of the study population

	Hospital (*n* = 14)	Home (*n* = 13)	*p*-value
Age (years)	55.64 (11.35)	56.50 (6.01)	ns
Sex Male n (%)	14 (100)	14 (100)	ns
Risk factors			
Hypertension n (%)	11 (78.57)	9 (64.28)	ns
Hyperlipidaemia n (%)	5 (35.71)	7 (50)	ns
Diabetes n (%)	1 (7.14)	6 (42.85)	0.029*
Overweight n (%)	8 (57.14)	5 (35.71)	ns
Obese n (%)	6 (42.85)	6 (42.85)	ns
BMI (kg/m^2^)	29.29 (3.62)	28.64 (3.65)	ns
Waist-hip ratio (cm)	106.42 (8.84)	104.14 (10.21)	ns
Current smokers n (%)	2 (14.28)	2 (14.28)	ns
Coronary artery disease			
Monovessel n (%)	3 (21.42)	8 (57.14)	ns
Double vessel n (%)	5 (35.71)	4 (28.57)	ns
Triple vessel n (%)	5 (35.71)	1 (7.14)	ns
Revascularization technique			
CABG n (%)	2 (14.3)	1 (7.1)	ns
PCI n (%)	11 (78.6)	11 (78.6)	ns
PCI & CABG n (%)	1 (7.1)	2 (14.3)	ns
Blood pressure			
Systolic blood pressure (mmHg)	115.35 (23.73)	119.35 (24.05)	ns
Diastolic blood pressure (mmHg)	69.28 (11.41)	71.50 (9.48)	ns
Exercise stress test			
Exercise time (min)	5.47 (2.08)	5.84 (2.66)	ns
METS	7.10 (1.97)	7.55 (2.77)	ns
Heart rate maximal (bpm)	132.84 (16.11)	106.21 (32.58)	0.02*
% of predicted peak HR.	79.86 (12.27)	65.10 (20.09)	0.02*
Hypertension response n (%)	5 (41.7)	4 (28.6)	ns
Clinical positive response n (%)	1 (7.1)	0 (0)	ns
Electrical positive response n (%)	2 (14.3)	0 (0)	ns
Ejection Fraction (EF)	52.33 (3.51)	51.00 (7.9)	ns
Laboratory values			
Total cholesterol (mg/dL)	130.50 (32.16)	145.71 (29.35)	ns
HDL cholesterol (mg/dL)	37.85 (8.07)	39.84 (9.21)	ns
LDL cholesterol (mg/dL)	67.17 (23.19)	73.68 (25.62)	ns
Triglycerides (mg/dL)	161.21 (72.31)	148.35 (58.16)	ns
Glucose (mg/dL)	94.92 (13.56)	111.00 (28.03)	ns
HbA1c	5.62 (1.05)	5.72 (0.93)	ns
Health-related quality of life			
SF-36 (score)	53.33 (19.80)	48.44 (20.89)	ns

Values are presented as mean (SD) unless stated otherwise

* Significance level *p* < 0.05

The right coronary and anterior descending arteries were the most affected among patients with either single-vessel disease or double-vessel lesions in both groups; while in patients with triple-vessel disease the affected arteries were either the anterior descending, circumflex, and marginal obtuse arteries, or the anterior descending, right coronary, and circumflex arteries. In general, the majority of patients underwent PCI, and 78.6% of the patients received pharmacoactive stents.

The medication prescribed to patients is similar in both groups (Table 3). Therapeutic doses were adjusted in each case to maintain heart rate, blood pressure and blood glucose levels within the recommended levels according to clinical practice guidelines for the control of cardiovascular risk factors in patients with heart disease Ischemic.

**Table 3:** Prescribed medication to patients

Medication	Hospital (*n* = 14)	Home (*n* = 13)
Antiplatelet agents	-	-
Acetylsalicylic acid	14	14
Clopidrogrel	3	5
Tricagrelor	8	5
Rivaroxavan	-	-
Plasugrel	2	4
Stomach protectors	14	14
Antiarrhythmics		
Bisoprolol	13	13
Carvedilol	1	-
Ranazolina	-	1
Antihypertensives	-	-
IECA	10	9
ARA-II	3	4
Diuretic	4	4
Amlodipino	4	1
Oral antidiabetic agents	1	7
Insulin	1	3
Anxiolytic	8	7
Statins	14	14
Hipnotic	2	2
Antidepressant	1	2
Epleronona	1	1

The exercise time and METS achieved during the exertion test as well as the recovery rate in the first minute increased in both groups after the intervention, with no significant differences being found between the usual cardiac rehabilitation group and the home-based with mixed surveillance group (Table 4).

**Table 4:** Measures results obtained before and after the two different cardiac rehabilitation programs

	Hospital based cardiac rehabilitation program		Home based cardiac rehabilitation program		*p*-value^a^	*p*-value^b^
	Before	After	Before	After		
METS	7.10 (1.97)	8.45 (1.71)	7.55 (2.77)	8.28 (2.62)	0.03*	0.49
Exercise time (min)	5.47 (2.08)	6.87 (1.67)	5.84 (2.66)	6.38 (1.80)	0.03*	0.32
Heart rate maximal (bpm)	132.84 (16.11)	123.38 (17.38)	107.23 (32.58)	123.84 (22.61)	**-**	0.009**
% of predicted peak HR.	79.86 (12.27)	75.16 (9.2)	65.10 (20.09)	75.81 (14.07)	**.**	0.008**
Borg Scale	12.57 (1.15)	13.57 (1.22)	13.07 (1.49)	13.69 (1.18)	0.015*	0.54
Recovery rate 1 min (bpm)	13.85 (5.02)	17.21 (6.29)	11.63 (7.41)	13.81 (4.46)	0.008**	0.54
BMI (kg/m^2^)	29.29 (3.62)	29.08 (37.6)	28.60 (3.80)	29.08 (4.33)	0.54	0.13
Waist-hip ratio (cm)	106.42 (8.84)	105.35 (9.96)	103.84 (10.56)	103.69 (10.59)	0.24	0.37
Total cholesterol (mg/dL)	130.50 (32.16)	138.21 (25.41)	142.66 (29.44)	135.91 (23.43)	0.91	0.10
HDL cholesterol (mg/dL)	37.85 (8.07)	20.57 (8.75)	39.45 (8.99)	40.45 (7.01)	0.24	0.58
LDL cholesterol (mg/dL)	62.17 (23.19)	68.62 (16.63)	70.30 (26.14)	61.13 (15.50)	0.74	0.06
Triglycerides (mg/dL)	161.21 (72.31)	161.07 (102.20)	149.83 (61.56)	160.08 (84.71)	0.77	0.76
Glucose (mg/dL)	94.92 (13.56)	104.07 (27.73)	109.83 (28.67)	106.75 (14.61)	0.57	0.26
HbA1c	5.62 (1.05)	5.75 (1.30)	6.05 (1.18)	6.15 (1.36)	0.35	0.90
Systolic blood pressure (mm Hg)	115.76 (24.65)	119.23 (19.45)	119.35 (24.05)	120.35 (16.69)	0.65	0.80
Diastolic blood pressure (mm Hg)	69.23 (11.87)	72.30 (11.65)	71.50 (9.48)	72.14 (11.21)	0.45	0.62
SF-36 (score)	53.33 (19.80)	63.63 (21.00)	47.93 (22.57)	43.62 (24.20)	**-**	0.004**

Data are presented as mean values (SD)

^a^Significance level for the hypothesis of no time effect

^b^Significance level for the hypothesis of no time x group effect (difference in outcome improvement between the two programs)

*Significance level *p* < 0.05

**Significance level *p* < 0.01

Significant values are highlighted in bold

The maximum heart rate reached during the stress test, however, appeared to be significantly differences between both groups, although it is not possible to affirm that the heart rate in any individual significantly changed (usual cardiac rehabilitation group: −17.57 [IC95%: −35.348, .205, *p* = 0.052]; home-based with mixed surveillance group 9.46 [IC95%: −.133, 19.056, *p* = 0.053]).

The only differences found between the two groups were in quality of life. The results obtained in the SF-36 post-intervention were significantly higher in the usual cardiac rehabilitation group (10.93 [IC95%: 17.251, 3.334, *p* = 0.007]) with no changes compared to the initial status in the home-based with mixed surveillance group (−4.314 [IC95%: −11.414, 2.787; *p* = 0.206]).

During the training sessions, two patients from the control group presented with angina-type pain without electrical changes evident on telemonitoring which subsided with rest, one patient presented an arrhythmia (*de novo* atrial fibrillation), two patients had a hypertensive response to the exercise which subsided with rest, and one patient had a hypotensive response which required the administration of intravenous saline solution.

In the experimental group, one patient had a hypertensive response to exercise which subsided during the cool-down phase, and one patient had a hypotensive response which required the intravenous administration of saline solution; both events occurred during the supervised training sessions carried out at the hospital.

No patient required treatment at the hospital’s Accident & Emergency (A&E) department.

The control group had 24 supervised training sessions in the hospital. Although it was recommended that the control group also exercise at home, we do not have a record of -home sessions. The experimental group’s patients completed an average of 36 sessions at home. The home-based training sessions lasted over an hour in 83.3% of cases. During the home-based exercise sessions supervised using the NUUBO® device, 8 patients pressed the alarm button in 20 training sessions, of which 3 patients reported angina-type pain during the exercise which subsided with rest; no ECG changes were seen on the recording in any of the cases. None of the other patients presented with angina-type pain or ECG changes during the home-based training sessions.

No serious cardiovascular complications were recorded during the cardiac rehabilitation programme.

## Discussion

The majority of patients who participate in cardiac rehabilitation programmes are middle-aged men. A number of studies have identified the patients who could benefit the most from cardiac rehabilitation and they include patients with significant functional deterioration, patients of advanced age, women, and ethnic groups, although these patients are the least likely to participate in cardiac rehabilitation programmes [1, 5]. Several studies have concluded that the effectiveness and safety of these home-based programmes could reduce inequality by improving access to this type of health service [20–23].

This study demonstrated that the cardiac rehabilitation with a mixed surveillance programme was as effective as the usual programme for improving functional capacity in moderate-risk patients.

Other studies have obtained similar results in low-risk patients after coronary revascularisation surgery and in patients of advanced age [8, 22, 24, 25]. While some of these studies analysed exercise capacity based on other parameters such as oxygen consumption, the shuttle walk test, or total cycle work capacity, the studies all found that home-based exercise programmes improved exercise capacity, and they found no differences when they compared them to hospital programmes [25].

When the training protocols for the different intervention models were compared, considerable variation was seen in the duration, frequency, and intensity of the exercise [25]. Despite these variations, all of the studies showed a beneficial short-term impact on exercise capacity after low-risk or moderate-risk patients completed a home-based cardiac rehabilitation programme. Furthermore, some of the studies demonstrated that long-term home-based programmes had better results than programmes carried out in hospitals.

The improved exercise time and recovery rate detected in the first minute also suggest an improved survival prognosis in patients with coronary heart disease [26]. All of these factors, together with the intensity of the exercise attained over the final weeks, could explain the increased subjective perception of the exertion measured using the Borg scale in both groups, and the improved risk profile of these patients.

With respect to the maximum heart rate achieved in the stress test, although the sample is not large enough to affirm that the changes observed in each separate group are statistically significant, it is sufficient to affirm the different effects of exercise on both groups. The decrease in heart rate in the stress test of the control group together with the increase in exercise time and the METs that were achieved, translate into a better adaptation to exercise than that of the experimental group. This is probably due to the fact that it was not possible to control caloric consumption per training session, the amount of time they remained at goal intensity during the -home exercise session, and walking speed, which are aspects that are related to improvement in prognosis and reduction in mortality [3, 4] and hospitalizations of these patients [2]. In this sense, the results obtained from this pilot study allow for the introduction of improvements in the design of these devices, which allow not only the monitoring of these patients, offering an efficient and safe way to exercise, but they also provide the patient enough flexibility to exercise anywhere with a workload that is adequate and easily modifiable [12].

The beneficial effect of exercise on lipids and the metabolism of carbohydrates has been documented in several studies, but in this study we found no significant differences when we compared the readings in both groups. The patients who participated in the study from the start presented with blood glucose, cholesterol and triglycerides readings within the normal ranges; only the HDL levels were below optimal levels. Other authors have also not seen improvements in HDL levels in patients with coronary heart disease [27]. In general, there is a high variability in results when the effect of exercise on the metabolism of lipids are analysed due to the heterogeneity of the study methods, duration of studies, populations, exercise protocols, and other contributory factors such as diet or lipid-lowering pharmacological agents. It is also known that in adults aged 50 and over, prolonged periods of exercise are needed to achieve a change in lipid metabolism, so the intervention duration of two months may not have been long enough to achieve an effect comparable to the results seen in other studies [28–30] on the lipid profile, body mass index, and abdominal circumference.

In terms of the quality of life, other studies have shown a significant improvement in both groups [30–34], and sometimes the effect was even greater in the home-based group [30]. However, in this study we detected an improvement in the usual cardiac rehabilitation group. The differences in the risk profile of the patients as well as other associated psychosocial factors may partially explain the results we obtained [35].

The exercise sessions at the hospital likely promoted social support due to the nature of the programmes and this could have impacted on patients’ perceived quality of life, as they feel like they were part of a group, with everything that entails [36]. It is be plausible that the exercise group in the traditional cardiac rehabilitation model achieved greater social cohesion and thus the programme had a greater impact on their quality of life.

The strategies which increased the frequency of exercise could in turn have contributed to a faster improvement in the perceived quality of life [30].

These and other factors, such as creating a home-based support network, will need to be taken into consideration when designing home-based cardiac rehabilitation programmes in order to achieve better results when it comes to the quality of life.

The safety of the exercise programme for moderate-risk patients was an important question, particularly when the programme was performed at home [37]. The safety of the exercise programme for patients with coronary heart disease has generally been related to the intensity and duration of the training and the patient selection. The individual risk assessment is a fundamental starting point here which serves as a guide for decision-making, among other things, when it comes to determining the right level of surveillance [11].

There are a wide variety of monitoring and surveillance strategies for home [9, 38, 39], but there are very few studies which use ECG monitoring systems through various devices [1, 12, 21, 22, 39–41]. Ades et al. [9] maintained that not all patients require transtelephonic monitoring in all sessions. Indeed, they suggested the selective use of this technology only in the initial sessions, and/or depending on the characteristics of each patient and their individual progress, probably because they did not differentiate between low and moderate-risk patients in their sample. However, Giallura et al. [21] maintained that telemonitoring improved compliance and the results of a home-based cardiac rehabilitation programme, particularly the functional capacity profile, compared with patients who participated in a home-based cardiac rehabilitation programme without ECG monitoring.

Bearing in mind the characteristics of the patients in this study, the mixed surveillance model described required, for the whole programme, the use of a telemetric monitoring system which allowed the information stored on the device during the exercise session to be sent from home so that it could be evaluated at the hospital without the patient needing to go to the centre. We have not found any other study which assessed a similar mixed surveillance model for the whole programme in moderate-risk patients.

Patients generally accepted this new exercise monitoring model away from the hospital, which made the telemonitoring system mixed surveillance exercise programme a viable option for moderate-risk patients.

However, this intervention model in higher-risk patients establishes a new scenario in which it is not only necessary to have a clear definition of the risks and responsibilities, but also the various clinical skills among the health professionals [10].

Other studies have demonstrated the safety of home-based cardiac rehabilitation programmes in lower-risk patients [23, 38, 39]. Bearing in mind that the complications recorded in the home-based group with mixed surveillance were minor, we believe that this model of intervention was safe in low-risk patients with coronary heart disease.

There are some limitations derived from the sample size. Although the sample size in the current study are small, this is a preliminary investigation regarding an alternative method for mixed-home supervision, which that could improve access to this type of program for cardiac patients with a risk-profile that is greater than has been previously reported. The study currently continues to recruit patients. Further, another limitation of the current study is that the sample only consists of men since none of the women who came in for an evaluation for the cardiac rehabilitation program met the inclusion criteria for taking part in this study. Additionally, with relation to heart disease, despite the randomization procedure described in the manuscript, the experimental group consisted of a majority of patients presenting with monovessel disease, while the majority of the control group had presented with two or three vessel disease. However, all the patients had stable ischemic heart disease and met the moderate risk criteria. Finally, although it was recommended that the control group exercise at home, we do not have a record of in-home sessions.

In spite of these limitations, the results of this study may help with the design of home-based programs that are adapted to different sociodemographic situations for moderate-risk coronary patients and also in the design of new technologies that are adapted to the profiles of these patients, which can improve access to Cardiac Rehabilitation services. However, the long-term effects need to be assessed for this patient population through multicentre studies to determine the effectiveness and acceptability of these mixed surveillance programmes based on telemedicine in different contexts. It is necessary to point out that the study is continuing with the added participation of different hospitals (multicentre study) and we will soon have the first results on long-term monitoring.

## Conclusion

A home-based cardiac rehabilitation programme with mixed surveillance was as effective and safe as the usual model in moderate-risk patients with ischaemic cardiomyopathy. However, the cardiac rehabilitation programmes carried out at the hospital had a greater impact on the patients’ quality of life. The results presented here suggest that the implementation of this programme would represent an improvement in the care quality of the public health services as it could offer benefits in the type and number of potential patients helped, the possibility of personalised treatment tailored to the different lifestyles or needs of patients, and thus enhance treatment adherence. Future lines of research should analyse how the design of home-based programmes could improve these results, bearing in mind the multidimensional nature of quality of life.

## Abbreviations

AMI: Acute myocardial infarction
AV: Atrioventricular
BMI: Body mass index
BP: Blood pressure
CABG: Coronary artery bypass grafting
CRT: Cardiac resynchronization therapy
ECG: Electrocardiogram
EF: Ejection fraction
HR: Heart rate
ICD: Implantable cardioverter-defibrillator
PCI: Percutaneous coronary intervention
RPE: Rating of perceived exertion
